# Magnetic Solid-Phase Extraction Based on Poly 4-Vinyl Pyridine for HPLC-FLD Analysis of Naproxen in Urine Samples

**DOI:** 10.3390/molecules25122924

**Published:** 2020-06-25

**Authors:** Karen A. Escamilla-Lara, Ana C. Heredia, Araceli Peña-Alvarez, Israel S. Ibarra, Enrique Barrado, Jose A. Rodriguez

**Affiliations:** 1 Area Academica de Quimica, Universidad Autonoma del Estado de Hidalgo, Carretera Pachuca-Tulancingo Km. 4.5, Mineral de la Reforma 42184, HGO, Mexico; qescamilla04@gmail.com (K.A.E.-L.); israel_ibarra@uaeh.edu.mx (I.S.I.); 2 Facultad de Quimica, Departamento de Quimica Analitica, Universidad Nacional Autonoma de Mexico, Ciudad de Mexico 04510, Mexico; hera21.ach@gmail.com (A.C.H.); arpeal@unam.mx (A.P.-A.); 3 Department of Analytical Chemistry, Facultad de Ciencias, Universidad de Valladolid, Campus Miguel Delibes, Calle Paseo de Belen, 7, 47011 Valladolid, Spain; ebarrado@qa.uva.es

**Keywords:** MSPE, HPLC-FLD, 4-vynilpiridine, naproxen

## Abstract

A magnetic solid phase extraction technique followed by liquid chromatography with a fluorescence detector for naproxen analysis in human urine samples was developed. The method includes the extraction of naproxen with a magnetic solid synthetized with magnetite and poly 4-vinylpriridine, followed by the magnetic separation of the solid phase and desorption of the analyte with methanol. Under optimal conditions, the linear range of the calibration curve was 0.05–0.60 μg L^−1^, with a limit of detection of 0.02 μg L^−1^. In all cases values of repeatability were lower than 5.0% with recoveries of 99.4 ± 1.3%. Precision and accuracy values are adequate for naproxen (Npx) analysis in urine samples.

## 1. Introduction

**Figure 1:**
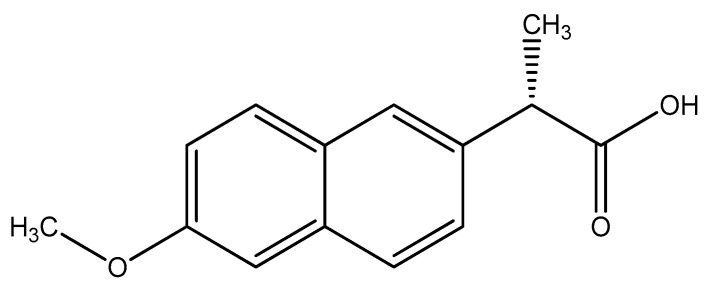
Naproxen (Npx) chemical structure.

Naproxen (Npx) is a pharmaceutical compound that belongs to the group of non-steroid anti-inflammatory (AINEs). Its properties allow it to act as anti-inflammatory, analgesic, and antipyretic [1], it is used for the treatment of rheumatoid arthritis, osteoarthritis, acute traumatic lesion and prophylactic treatment [2]. Structurally it is a derivative of propionic acid (Figure 1), shows values of log P 3.18 and pKa 4.2. Its main function is to inhibit the production of cyclooxygenase and reduce the concentration of prostaglandin in several biological fluids and tissues [3].

There are different techniques described to quantify Npx, such as: UV-Vis spectroscopy [4], gas chromatography [5], liquid chromatography [6], and capillary electrophoresis [7]. The aforementioned techniques require a previous sample preparation step because of pharmaceutical compound is found in concentrations of µg L^−1^ or ng L^−1^ in different analytical matrices [8,9].

Solid phase extraction (SPE), using octadecylsilane (C18) or polymeric cartridges (*N*-vinylpyrrolidone and divinylbenzene) has been preferred for the preconcentration process of Npx [10,11]. Solid phase microextraction techniques were also proposed in order to decrease the amount of solvents and its limits of detection achieved when it is coupled to sensitive instrumental techniques [12,13]. Another alternative is dispersive solid phase extraction, d-SPE [14], which it is characterized by the use of adsorbent materials in nanometric scale improving the contact between the adsorbent surface and the analyte [15]. The extraction phase should be modified to improve selectivity, however, nanomaterial separation is complex. The incorporation of a paramagnetic phase to the adsorbent facilitates the separation using an external magnetic field, avoiding filtration or centrifugation steps. This d-SPE variation is co-called magnetic solid phase extraction (MSPE) [16]. The adsorbents employed are commonly composed by a magnetic core, usually magnetite (Fe_3_O_4_), covered by a shell which promoted selectivity in the extraction process and protect the magnetite from degrading. Some solids proposed for Npx analysis includes a shell phase of, carbon (graphene and carbon nanotubes), silica, and polymer [17,18,19,20,21,22,23].

Organic polymers have shown to be a viable alternative to protect magnetic core, furthermore they allows the incorporation of a large variety of functional groups in the surface [24]. Npx is an acid molecule and therefore the presence of an aromatic monomer of basic nature promotes the adsorbent–analyte interaction via acid–base or π–π interactions [25,26]. This work proposed the synthesis of a magnetic solid based on poly-4-vinyl pyridine (P4-VP).

## 2. Results and Discussion

### 2.1. Chemical Characterization of Magnetics Solids

**Figure 2:**
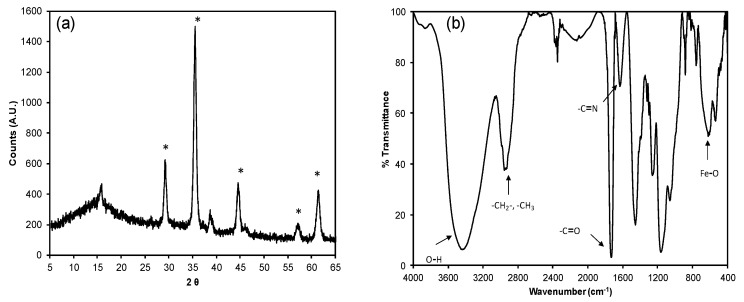
(**a**) Diffraction pattern for the magnetic solid and (**b**) FTIR spectrum for solid C. * Fe_3_O_4_.

The diffraction spectra in all samples show a similar profile, Figure 2a shows the X-ray diffraction pattern for the magnetic solid C (see Section 3.3). A wide peak at small angles, around 15° is related to the presence of amorphous polymeric material. A crystalline phase is identified at angles 2θ: 30.1°, 35.5°, 43.1°, 53.4°, and 62.6°, which corresponds to Fe_3_O_4_ [27]. The FTIR analysis (Figure 2b), shows a vibration band characteristic of the Fe–O bond at 571 cm^−1^, the signals between 3250–3750 cm^−1^ belong the stretching of the O–H bond [28], at 2960 cm^−1^ the vibration of *−*CH_2_*−*, *−*CH_3_ is observed, around of 1730 cm^−1^ is the band attributed to carbonyl group contained within the EGDMA and MA monomers, at 1654 cm^−1^ the vibration of the stretching of the –C=N bond is observed, characteristic of the pyridine ring [29].

### 2.2. Adsorption Capacity of Magnetic Solids

**Figure 3:**
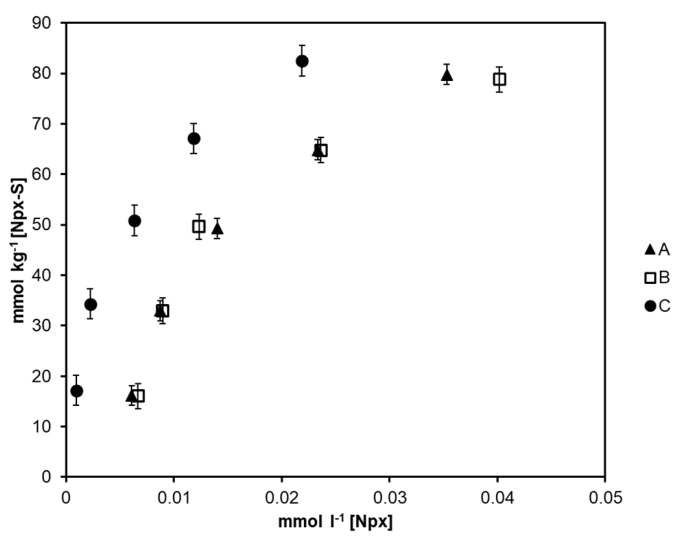
Adsorption isotherms for the three synthesized magnetic solids. Experimental conditions: room temperature, pH value of 2.0 (adjusted with HCl) and contact time of 30 min.

Adsorption was evaluated a pH values of 2.0, 4.0, and 6.0, however, Npx was not retained at pH > 4.0. The Figure 3 shows the adsorption isotherms (at pH = 2.0) for the three synthesized magnetic solids. The value of the affinity constant among magnetic solids is estimated and the analyte used in the scatchard graph adjusts to Equation (1), where [Npx–S] (mmol kg^−1^) corresponds to the Npx adsorpted on the solid and [Npx] (mmol L^−1^) is the concentration of the pharmaceutical compound in liquid phase after adsorption process. Data are adjusted to a straight line according to Equation (1) where Qmax is the maximum quantity of Npx that can be adsorbed and Kd is the affinity constant estimated from the slope (−1/Kd) for the equilibrium Npx–S ⇆ Npx + S.

(1) $$\frac{\left[\mathrm{Npx}\;-\;S\right]}{\left[\mathrm{Npx}\right]}=\frac{Q_{\max}}{K_{d}}\;-\;\frac{\left[\mathrm{Npx}\;-\;S\right]}{K_{d}}$$

Table 1 shows the Kd values found, in all the cases the estimated value is located at the 1 × 10^−7^ and 1 × 10^−4^ M interval, which is appropriate for the retention and elution processes [30]. From the three solids, C shows more affinity towards Npx. The values estimated are congruent to the number of active groups determined (expressed as mmol HCl kg^−1^). At low pH values, magnetite [31] and P4-VP [32] surfaces are positive charged, in consequence repulsion force favored the exposition of pyridinium group. In this sense, magnetite content is higher in the solid (C) and in consequence, an increment on active sites and analyte–solid affinity was observed. The results showed a moderate adsorption capacity because of the low molar ratio of 4-VP, however an increment on concentration of functional monomer produces solubility of P4-VP decreasing protection of magnetic core and adsorption capacity.

### 2.3. Optimization of the MSPE Conditions

A retention–elution process is affected by several variables. Taguchi experimental design was selected as a robust optimization method to evaluate the control variables involved on MSPE [33]. In the separation using MSPE, the output signal is maximizing the response (Area _Npx_/Area _I.S_). The control variables involved in the process are: composition of the magnetic solid, [NaCl] added (M), pH value, and methanol volume (mL) employed for elution [34]. Three levels for each factor were selected using an orthogonal matrix L_9_(3^4^). The tests were performed using a spiked urine sample (50.0 µg L^−1^) of Npx. The design matrix and the results are shown in Table 2.

**Figure 4:**
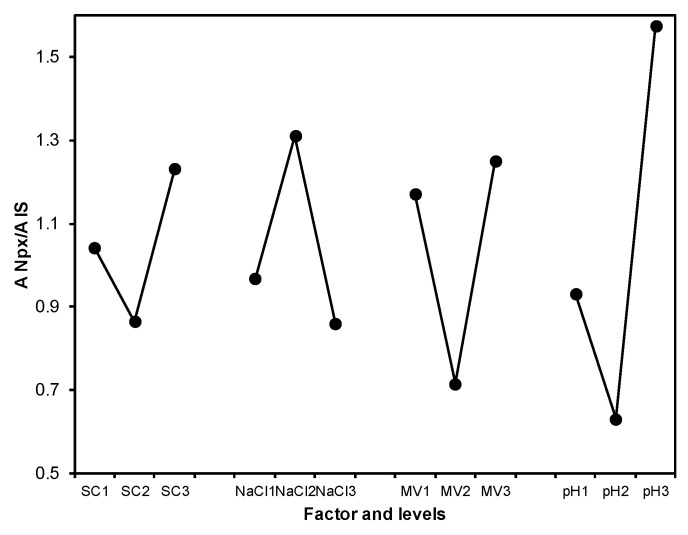
Effect of the control factors over the Npx extraction process.

Figure 4 shows the effect of the factors over the output variable, being the pH the factor with highest contribution to the system (41.11%), followed by the methanol volume (23.3%), [NaCl] added (19.64%), and finally the composition of the magnetic solid (15.99%).

Based on the adsorption results, it is confirmed that the most adequate solid is the solid C. Additionally, the magnetite concentration is higher which promotes a better recovering of the magnetic solid. An increase on the ionic force reduces the solubility of the hydrophobic compounds promoting the retention of the analyte on the extractive phase [35]. The results indicate that increase of NaCl concentration promotes Npx retention. However, when [NaCl] is employed 1.5 M there is a competence between chlorine ions and the analyte during adsorption, affecting retention [36]. Since [NaCl] of 1.0 M yielded higher signals, it was chosen as optimal concentration value.

Regarding the pH value, this variable is important in extraction processes of substances with acid-basic properties. The extraction of Npx is improved when the pharmaceutical compound is in its neutral form, adjustment of the pH value should be performed below the Npx pKa value. The optimal value found was pH = 3.0, P4-VP is positive charged (pyridinium cation) at this pH value. However, the multiple aromatic units covalently attached on the adsorbent can interact with planar aromatic analytes [37]. This result indicates that presence of naphthalene ring in naproxen structure increased interaction with P4-VP via π–π interaction.

The elution solvent was selected considering the polarity (less polar than the analytical matrix) and the solubility of analyte. Several methodologies showed that methanol is adequate eluent for Npx because of solubility of the pharmaceutical compound and additionally it is a component of the mobile phase [38]. A volume of 3.0 mL of methanol was selected for Npx elution.

### 2.4. Method Validation and Urine Samples Analysis

**Figure 5:**
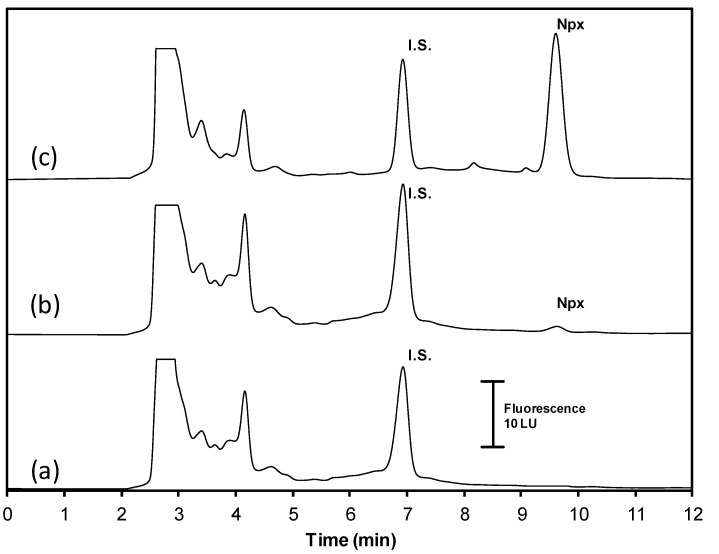
Chromatogram obtained from the analysis of an urine sample under optimal conditions. (**a**) blank sample, (**b**) Npx 0.05 µg L^−1^, (**c**) Npx 35 µg L^−1^. Internal standard (I.S. 50 µg L^−1^).

Under the following opimal conditions: solid C, NaCl concentration 1.0 M, pH = 3.0, and 3.0 mL of methanol, it was constructed the calibration curve using urine spiked standards of 5.0, 10.0, 20.0, 30.0, 40.0, 50.0, and 60.0 µg L^−1^ of Npx. The calibration curve was adjusted to the following equation: A_Npx_/A_IS_ = 14.868 ± 0.75 [Npx]/[IS] + 0.0182 ± 0.06 (R^2^ = 0.99) with a linear range of 0.05–60.0 µg L^−1^ Limit of detection and limit of quatification were calculated according to FDA criteria [39] as 3.3 s_c_/b1 and 10 s_c_/b1, where s_c_ is the square root of the residual variance of the standard curve, and b1 is the analytical sensitivity. Limit of detection and limit of quatification were 0.02 µg L^−1^ y 0.05 µg L^−^^1^, respectively. In order to validate the limit of quantification, a spiked urine sample (0.05 µg L^−1^) was analyzed in triplicate and the data obtained were: a mean recovery of 104.1% with precision of 6.9% (expressed as relative standard deviation, *n =* 3). Figure 5 shows a chromatogram obtained for analysis of: blank urine sample (Figure 5a) and spiked with 0.05 (Figure 5b) and 35 µg L^−1^ (Figure 5c) of Npx. Absolute recovery was evaluated by comparing the signal ratio obtained from the analysis of a Npx standard (10.0 µg L^−1^) prepared in urine and deionized water, by the proposed methodology. An absolute recovery value of 83.5% was obtained as a consequence of co-extraction of urine components.

Precision was evaluated in terms of within-day and between-days repeatability for calculated Npx concentration, determined by interpolation in calibration curve obtained with spiked urine standards. Results were expressed as relative standard deviation (% RSD) obtained from the analysis of spiked urine samples (*n =* 3) with Npx concentrations of 5.0, 30.0, and 50.0 µg L^−1^ during three days, in all cases the values were lower than 5.0%. Accuracy of the proposed method was investigated by determining the recovery of Npx in spiked urine samples. Recovery was expressed as % Recovery = (100 [Npx]_calculated_)/[Npx]_added_. The results are summarized in Table 3 and indicate acceptable recovery factors.

MSPE-HPLC-Fluorescence detection methodology was applied to analyze Npx in four different urine samples (two men and two 2 women), Npx concentration was determined by interpolation into the calibration line obtained with spiked urine samples. Initial concentrations were in the interval of 1.3–8.2 µg L^−1^. In order to evaluate applicability, urine samples were spiked with 15 and 35 µg L^−1^ of Npx and they were analyzed in triplicate. Results obtained are shown in Table 4, the average recovery was 98.6 ± 1.6%. Considering the differences in the matrix composition and the presence of creatinine, inorganic salts, urea, uric acid, the methodology proposed is useful for analysis of Npx in urine samples.

Table 5 shows a comparison between the results obtained by the proposed method and others proposed for Npx determination in human urine. The proposed method has competitive limits of detection with the advantage of reducing the analysis time and decrease sample and eluent volumes.

## 3. Materials and Methods

### 3.1. Chemicals

Ethylene glycol dimethacrylate (EGDMA), (S)-(+)-6-Methoxy-α-methyl-2-naphthaleneacetic acid (Npx), methyl acrylate (MA), methanol HPLC grade, 4-vynilpiridine (4-VP), and 1-naphtaleneacetic acid (internal standard, IS) were obtained from Sigma Aldrich (St Louis, MO, USA). Sodium chloride, ammonium persulfate, ferrous sulfate heptahydrate, sodium hydroxide, polysorbate 80 (Tween 80), acetic acid and hydrochloric acid were purchased from J.T. Baker (Phillipsburg, NJ, USA). All the solutions were prepared using deionized water from Milli-Q system with a resistivity no less than 18.0 MΩ (Millipore, Bedford, MA, USA).

### 3.2. Equipment

The characterization of the synthesized solids was made by using an INEL Equinox 2000, diffractometer with CoKα1 radiation (λ = 1.7890100 Å). The patterns were recorded in a 2θ interval of 20-70° with increments of 0.02° (2θ). The pH measurements were performed with a pH meter Oakton pH510 Series (Vernon Hills, IL, USA). For the infrared spectroscopy study, a Perkin Elmer spectrometer model GX (Waltham, MA. USA) was used in a range of 4000–400 cm^−1^, the samples were prepared and analyzed in KBr.

Npx analysis was performed by HPLC using an Agilent Technologies 1260 Infinity (DE, Germany) equipped with an Agilent Zorbax ODS Analytical 4.6 × 250 mm, 5-µm column. The analytic signals were detected using a fluorescence detector set at excitation and emission wavelengths of 271 and 356 nm, respectively. The mobile phase is comprised of 70% methanol and 30% of acetic acid aqueous solution at 1% (*v*/*v*) with a flow rate of 0.8 mL min^−1^. The injection volume was 20.0 µL. The equipment is controlled by the software Agilent OpenLAB.

### 3.3. Synthesis of Magnetic Solids

Three magnetic solids (A, B and C) were synthesized in two steps using the proportions shown in Table 6. The first step consists in the synthesis of the magnetite core from FeSO_4·_7H_2_O. NaOH was added to Fe (II) solution to reach a pH value of 10.0 ± 0.2 in presence of an air current, the ferrous precipitate was partially oxidized and separated from the liquid phase placing a magnet on the external part of the flask [42]. The solid was rinsed (25.0 mL ×5) with deionized water and the magnetic phase obtained was dispersed in 100 mL of Tween 80 (0.5% *w*/*v*) and it was stirred for 10 min at 75 °C.

The extracting phase was synthesized by emulsion polymerization adding 4-VP as functional monomer, EGDMA as crosslinker and MA. The monomers were selected because of solubility of P4-VP in water at low pH values, in order to protect the magnetic core and minimize loss of extracting phase, it was included MA and EGDMA to promote the synthesis of a hydrophobic crosslinked co-polymer [43]. The monomers were added to the magnetite suspension in the following order: MA, EGDMA and 4-VP. Once added, 5.0 mL of a solution of ammonium persulfate (1.4% *w*/*v*) is added and the mixture was stirred for an hour. The solid obtained was separated magnetically, rinsed with deionized water and dried at 60 °C for 12 h. The dry solids were pulverized in agate mortar and stored in dark until their use.

### 3.4. Characterization of the Solid

The number of active sites of each magnetic solid was determined using an acid titration. 50.0 mg of the magnetic solid were mixed with 10.0 mL of NaOH (0.01 M), the suspension was titrated then using HCl 0.01 M to achieve a pH value of 3.0, potentiometric recording showed two equivalence points. Active sited were determined by difference between equivalence points, and the value is expressed as mmol_HCl_ kg^−1^.

The adsorption capability of each solid was evaluated using adsorption isothermals at pH = 2.0 (adjusted with HCl 0.1 M). Under these conditions Npx is retained after reaction of the carboxylic group with P4-VP. Adsorption experiments were performed by mixing, 10.0 mg of each solid and 2.0 mL with Npx aqueous solution (20.0–100.0 mg L^−1^) in polypropylene tubes. The solutions were stirred during 30 min, the solid was magnetically isolated and the liquid phase was analyzed by HPLC.

### 3.5. Sample Analysis

**Figure 6:**
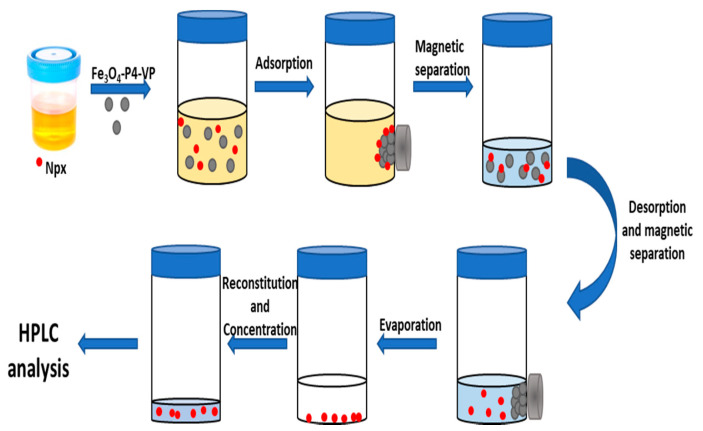
Schematic procedure for the isolation of several Npx from urine samples by magnetic solid phase extraction (MSPE).

Four urine samples (10 mL) from healthy individuals were stored at −20 °C until their analysis. The proposed methodology (Figure 6) involves the following steps. a) 10.0 mg of the magnetic solid was activated with 1.0 mL of methanol, it is washed twice with 1.0 mL of deionized water; b) 1.0 mL of the sample is diluted to 10.0 mL with a solution composed of HCl (1 × 10^−3^ M), NaCl (1.0 M) and the internal standard (50 µg L^−1^); c) the sample diluted was added to the activated magnetic solid and the mixture was mechanically stirred for 30 min; d) magnetic phase is separated using a neodymium magnet reserving the solid phase; e) the solid phase was washed with 1.0 mL of deionized water followed by 3.0 mL of methanol to eluted the analyte; f) the phases were magnetically separated and the liquid phase was evaporated, the residue was reconstituted in 0.5 mL of methanol and analyzed by HPLC.

## 4. Conclusions

In this work, solids based on magnetite and P4-VP were synthesized for their application in the separation of Npx in urine samples. The increment in the concentration of magnetite favors the disposition of the pyridine groups, improving the solid–analyte affinity. The methodology developed for the determination of Npx is robust, simple and fast, it uses small sample and solvent volumes, in addition to be a promising technique for the analysis of Npx in complex samples. The achieved detection limits are competitive with the ones described in other described methodologies.

## Figures and Tables

**Table 1:** Calculated parameters for the characterization of magnetic solids.

Solid	Kd (×10^−6^ M)	Active Sites (mmoL H^+^ kg^−1^)	Qmax (mmoL kg^−1^)
A	24.5 ± 0.1	700 ± 3	79.8 ± 1.0
B	20.4 ± 0.1	500 ± 2	78.8 ± 5.7
C	3.5 ± 0.1	1200 ± 4	82.5 ± 1.4

**Table 2:** Orthogonal matrix and its correspondent [Area Npx]/[Area SI] for each experiment.

Experiment	SC	NaCl (M)	MV (mL)	pH	[Area Npx]/[Area IS]
1	A	0.5	1	1	0.97
2	A	1.0	2	2	0.56
3	A	1.5	3	3	1.58
4	B	0.5	2	3	0.98
5	B	1.0	3	1	0.82
6	B	1.5	1	2	4.90
7	C	0.5	3	2	0.94
8	C	1	1	3	2.15
9	C	1.5	2	1	0.60

SC-solid composition, MV-methanol volume.

**Table 3:** Npx concentration determined in spiked urine sample using the proposed method.

[Npx]_added_ (µg L^−1^)	[Npx]_calculated_ (µg L^−1^)	Repeatability, Within-Day (%RSD, *n* = 3)	Repeatability, Between-Days (%RSD, *n* = 3)	% Recovery
5.0	4.9	2.2	3.7	98.0
30.0	30.2	1.6	1.9	100.7
50.0	49.7	1.5	2.1	99.4

**Table 4:** Npx concentration determined in urine sample using the proposed method.

Sample	[Npx]_added_ (µg L^−1^)	[Npx]_calculated_ (µg L^−1^, %RSD, *n =* 3)	[Npx]_total_ (µg L^−1^) ^a^	% Recovery ^b^
M1		8.2 (1.9)		
	15.0	22.1 (1.7)	23.2	95.3
	35.0	42.7 (1.3)	43.2	98.8
M2		7.8 (2.0)		
	15.0	23.1 (2.0)	22.8	101.3
	35.0	42.0 (0.7)	42.8	98.1
W1		6.0 (2.5)		
	15.0	20.3 (2.3)	21.0	96.7
	35.0	40.7 (2.7)	41.0	99.3
W2		1.3 (4.6)		
	15.0	16.4 (1.9)	16.3	100.6
	35.0	35.7 (1.3)	36.3	98.3

M—men, W—woman; ^a^ [Npx]_total_ = [Npx]_added_ + [Npx]_calculated_; ^b^ Recovery = (100 [Npx]_calculated_)/[Npx]_total._

**Table 5:** Comparison between methods used to determine Npx in human urine.

Sample Preparation Method	Detection System	LOD (µg L^−1^)	RSD (%)	Sample Volume (mL)	Reconstitution Volume (mL)	Reference
MMOF-SPME ^a^	HPLC-UV	0.03	<4.7	5.0	2.0	[40]
LDH-PS-μSPE ^b^	HPLC-UV	5.0	<7.4	0.25	0.2	[8]
MFA-SPE ^c^	HPLC-UV	2.0	<6.7	50.0	5.0	[41]
MIP-SPE ^d^	FLD	2.0	<1.0	40.0–100.0	2.0	[3]
MSPE	HPLC-FLD	0.02	<5.2	1.0	0.5	This work

^a^ Magnetic metal organic frameworks nanocomposite fiber solid-phase microextraction; ^b^ Micro-solid phase extraction by packed sorbent layered double hydroxide; ^c^ Magnetic field assisted µ-solid phase extraction; ^d^ Molecularly imprinted polymer solid-phase extraction.

**Table 6:** Molar ratio of the synthesized magnetic solids.

Solid	Magnetite (mmoL)	4-VP (mmoL)	MA (mmoL)	EGDMA (mmoL)
A	3.0	0.3	0.3	5.0
B	6.0	0.3	0.3	5.0
C	12.0	0.3	0.3	5.0
